# Genome-wide linkage analysis of systolic blood pressure: a comparison of two approaches to phenotype definition

**DOI:** 10.1186/1471-2156-4-S1-S13

**Published:** 2003-12-31

**Authors:** Susan L Slager, Stephen J Iturria

**Affiliations:** 1 Division of Biostatistics, Department of Health Sciences Research, Mayo Clinic, 200 First Street SW, Rochester, Minnesota 55905 USA; 2 Eli Lilly and Co., Indianapolis, Indiana 46285 USA

## Abstract

Problem 1 of the Genetic Analysis Workshop 13(GAW13) contains longitudinal data of cardiovascular measurements from 330 pedigrees. The longitudinal data complicates the phenotype definition because multiple measurements are taken on each individual. To address this complication, we propose an approach that uses generalized estimating equations to obtain residuals for each time point for each person. The mean residual is then taken as the new phenotype with which to use in a variance components linkage analysis. We compare our phenotype definition approach to an approach that first reduces the multiple measurements to a single measurement and then models these summary statistics as regression terms in a variance components analysis. For each approach, multipoint linkage analysis was performed using the residuals and the SOLAR computer program. Our results show little difference between the methods based on the LOD scores.

## Background

The phenotype definition is an important component of gene mapping. In gene mapping studies, each individual is assigned a phenotypic value for a particular trait of interest. This phenotype is then tested for cosegregation with certain markers. If a susceptibility locus exists, then individuals with similar trait values should have higher-than-expected allele sharing at this locus.

The data from the Framingham Heart Study complicates the phenotype definition in that up to 21 measurements over a 40-year time span were taken from each person (Cohort 1) or five measurements taken over a 20-year time span (Cohort 2). Moreover, these multiple measurements from a person are correlated with each other. Thus, it is unclear as how to define a phenotype given the correlated repeated measures. One approach would be to model each time point in a regression analysis and then combine the parameter estimates [1]. Another approach would be to model all the time points in a regression analysis, i.e., a multivariate response outcome [2]. This approach might be difficult to apply to the Framingham data because the number of time points in Cohort 1 (21 measurements) result in a large number of parameters to estimate. A third approach would be to reduce the multiple measurements from each person to a single measurement, thus eliminating the problem of correlated measurements within a subject. This is the approach taken by Levy et al. [3]. In their analysis, they first found the average systolic blood pressure (SBP), average age, and average body mass index (BMI) for each person. They then regressed the average SBP on the average age and average BMI. The residuals from this linear regression analysis were used as the phenotype in a quantitative linkage analysis. We applied an alternative summary-statistic approach. First, we modeled SBP on the covariates using a longitudinal analysis method and then averaged the residuals across the multiple measurements for each person. The average residual was then the individual's phenotype that was used in the linkage analysis.

We hypothesize that our approach will provide more linkage information because all of the data, rather than summary statistics, are used to estimate the parameters in the regression model. The approach of Levy et al. does use all of the data, but by averaging the variables first, information about variability is lost. We test this hypothesis by comparing the genome-wide linkage results using our phenotype-definition approach with the genome-wide linkage results using the phenotype-definition approach of Levy et al.

## Methods

### Data

Problem I data consists of 2885 individuals from 330 pedigrees. Of the 2885 individuals, 1213 were from Cohort 1. Cohort 1 individuals were followed every 2 years for a total of 21 measurements. Cohort 2 individuals are offspring of Cohort 1 individuals, and they were followed every 4 years for a total of five measurements. At each follow-up, extensive amount of medical information was obtained, including SBP, age, height, weight, and high-blood pressure treatment information.

### Longitudinal analysis

We are interested in finding the genes that increase the risk for cardiovascular (CV) disease. We used SBP as a surrogate to CV. We first analyzed the longitudinal data following the methods of Levy et al., denoted as Method 1. Specifically, we calculated the mean SBP, , for the *i*^th ^individual. We then used linear regression to regress  on ( - ) and ( - ), where  is the mean age of an individual,  is the mean BMI of an individual,  is the sample mean age, and  is the sample mean BMI. The residuals from this regression analysis are then used as the quantitative phenotype for the linkage analysis.

We also analyzed the longitudinal data with an alternative approach (Method 2). We first found the residuals for each time point. We then calculate the average residual over all time points for each person to use as the phenotype in the linkage analysis. To calculate the residuals, we used generalized estimating equations (GEE) [4]. This is a linear models approach that accounts for the dependency among the time points. We used an exchangeable working correlation matrix in the analysis and regressed SBP on age and BMI at the *i*^th ^time point. Residuals were then obtained for each time point.

For both approaches, we analyzed the phenotype data from males and females and from Cohorts 1 and 2 data separately, resulting in four longitudinal analyses. This was done to allow for different rates of change for age and BMI for each of the male/female and Cohort 1/Cohort 2 combinations. The residuals obtained from each of these four analyses were combined into one set of residuals, which was then used in the linkage analysis. The correlation between the two phenotypes used in the linkage analysis is 0.97.

### Linkage analysis

Multipoint linkage analysis of the residuals were completed using a variance-component approach, which tests for linkage by testing whether the variance component associated with a particular chromosomal location is significantly greater than zero. The analyses were performed using SOLAR [5]. Since we adjusted for age and BMI in the longitudinal analysis and ran separate longitudinal analyses for each sex and cohort combination, these effects were not modeled in the linkage analysis.

## Results

**Figure 1:**
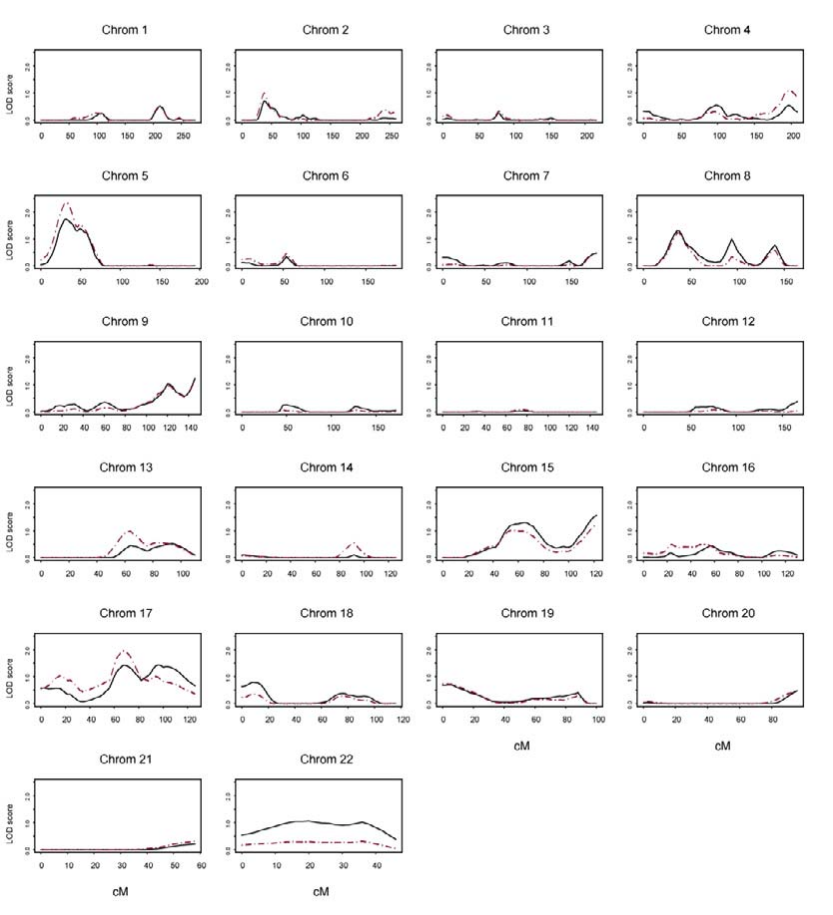
**Results from a genome-wide scan of standardized residuals of SBP. **Dotted lines denotes results from Method 1 phenotype analysis; solid line denotes results from Method 2 phenotype analysis.

Results of the linkage analyses from the two different approaches are presented in Figure 1. Multipoint LOD scores are plotted against chromosomal location for all 22 autosomes. From this figure, we see that the two analytical methods had similar results. This finding is also supported by the high correlation () between the two phenotypes. Moreover, neither of these methods reported LOD scores above 3. The largest multipoint LOD score that was observed was on chromosome 5, with a LOD score of 2.35 (Method 1) and a LOD score of 1.73 (Method 2) at 32 cM. These results were close to the result found by Levy et al.; they reported a multipoint LOD score of 1.9 at 23 cM.

Levy et al. found significant evidence for linkage (LOD score = 4.7) at 67 cM on chromosome 17. Although we did not achieve that level of significance with either approach (LOD score of 1.96 and 1.43 at 68 cM for Methods 1 and 2, respectively), we do see a peak at that location.

## Discussion

We present an alternative approach to deriving a phenotype from longitudinal data based on the GEE methodology, which accounts for the repeated measures from each observation. We hypothesize that our approach would provide more linkage information than that of Levy et al. because our approach uses all of the data to estimate the parameter estimates in the regression model. The approach of Levy et al. averages the longitudinal data first, thereby reducing the variability of the data, and then models the summary statistics in a regression analysis. What we observed, however, was that the approaches provided essentially the same amount of genetic information based on how similar the LOD scores are across the genome and how correlated the two phenotypes were. The two regions that had LOD scores of about 1.5 or higher occurred on chromosomes 5 and 17. At both regions, Method 1 had a LOD score that was about 0.55 units greater than that of Method 2. For the other regions, the results were similar or inconclusive, such as that found on chromosome 22. Since the maximum LOD score was about 1 on chromosome 22, no conclusions can be made about the difference between the methods in this region. This is because of the strong potential of false positives with such a low LOD score. The overall similarity between the methods indicates that little or no loss of information occurs by reducing the multiple measurements from each person to a single measurement before adjusting for other covariates.

A limitation of our study is that we use the real data to compare the two statistical methods. A more accurate comparison should be made with the use of simulated data, in which the true gene locations are known *a priori*. With simulated data, empirical type I error rates and power can then be determined for both methods, but a simulation study such as this requires at least 1000 replicates to appropriately test at the 5% significance level; the simulated data from GAW had only 100 replicates. We suspect that the conclusions drawn from the use of the simulated data (with 100 replicates) would not have been much more accurate than what we observed from the used of the real data. Another limitation of our study is that our approach (Method 2) did not account for familial relationships in the analysis. An assumption of GEE is independence among subjects, and a violation of this assumption may bias parameter estimates. We do observe a difference in parameter estimates between Cohorts 1 and II. This difference could be due to the fact that Cohort 2 consists of related subject, due to the fact that more data are available in Cohort 1, or it even could be due to ascertainment differences between the cohorts. However, based on Figure 1, a violation of this independence assumption of GEE does not appear to be a problem because both Method 1 and 2 had essentially the same LOD scores across the genome.

We note that our results from Method 1 differed from the published results of Levy et al. Even though our Method 1 was similar to that of Levy et al., our analysis method was not as extensive as theirs, e.g., Levy et al. accounted for treatment effects of hypertension, and they had inclusion criteria specifying which subjects to include in the analysis. In contrast, we included all subjects who had both phenotype and genotype information. Moreover, the Levy et al. analysis had two additional pedigrees. Thus, we expected the two results to differ. However, we emphasize that the primary goal here was to compare two analytical approaches rather than replicate the findings of Levy et al. In summary, since the two approaches provided similar results, we conclude that Method 1 is the more parsimonious approach to use because it requires fewer assumptions in the data analysis.
